# Supplementary material for: Neuroprotective Parkinson’s Disease Therapeutic: Transition Metal Dichalcogenide Nanoflower Treatments Alleviate Pathological Cell Stress
Source: bioRxiv. 2025 Oct 1:2025.09.29.679305. Preprint. [Version 1] doi: 10.1101/2025.09.29.679305 (PMC12621851; doi:10.1101/2025.09.29.679305)
Supplement: Supplement 1 [file media-1.pdf]

Neuroprotective Parkinson's Disease Therapeutic: Transition Metal Dichalcogenide Nanoflower Treatments Alleviate Pathological Cell Stress

Charles L. Mitchell[1,2], Mikhail Matveyenka[2], Harris C. Brown[2], Jessica Aldape[2], Payton Moore[2], Kha-Tran Nguyen[2], John C. Walker[2], Bryce Pearson[2], Joshua Skrehot[2], Dmitry Kurouski[1,2*]
[1] Interdisciplinary Program in Genetics and Genomics, Texas A&M University, College Station, Texas, USA
[2] Department of Biochemistry and Biophysics, Texas A&M University, College Station, Texas, USA

Email: dkurouski@tamu.edu

Supporting Information

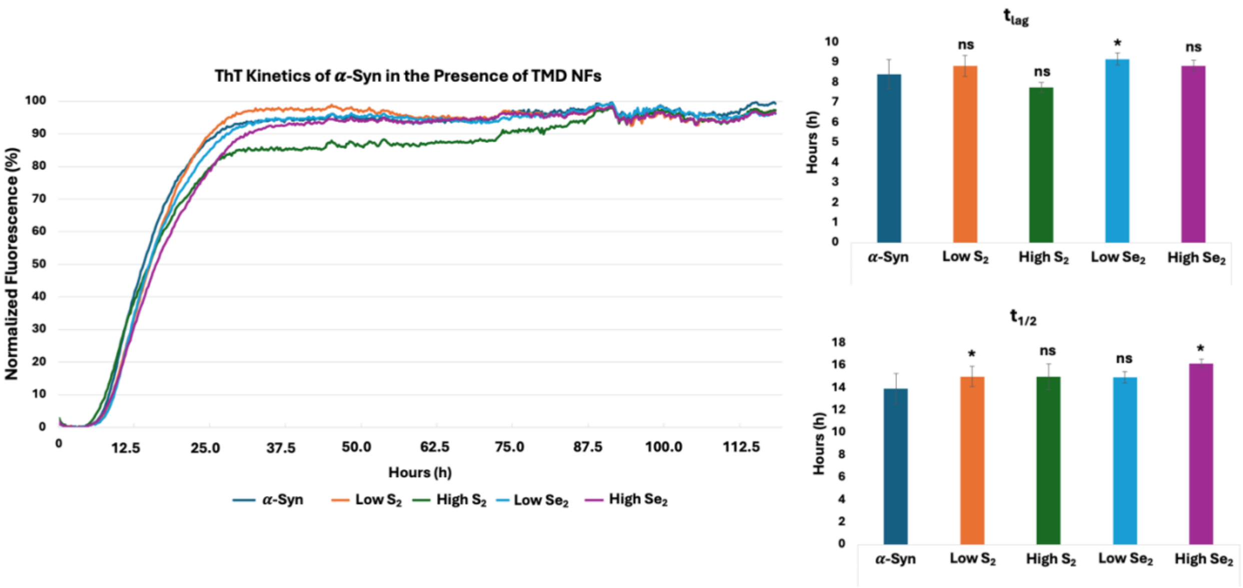

Figure S1: Aggregation kinetics for α-syn protein in the presence of TMD NFs at 0.1 mg/mL (low) and 1.0 mg/mL (high) concentrations via fluorescence of thioflavin T (ThT) [left]. Calculated t_{lag} [top right] and $t_{1/2}$ [bottom right] from the aggregation curves for α-syn in the presence of 0.1 mg/mL MoS_2 (Low S_2), 1.0 mg/mL MoS_2 (High S_2), 0.1 mg/mL $MoSe_2$ (Low Se_2), 1.0 mg/mL $MoSe_2$ (High Se_2), and the absence of NFs (α-Syn). Significance marking coincide with comparisons made to the α-syn control.
